# Changes in Shoulder Girdle Muscle Activity and Ratio During Pilates-Based Exercises

**DOI:** 10.3390/life15020303

**Published:** 2025-02-14

**Authors:** Seong-Ik Seo, Eui-Young Jung, Woo-Lim Mun, Su-Yeon Roh

**Affiliations:** 1 Department of Health Science, Gachon University Graduate School, Incheon 21936, Republic of Korea; 05ssi0928@gachon.ac.kr (S.-I.S.); noel950@gachon.ac.kr (E.-Y.J.); 2 Department of Exercise Rehabilitation, Gachon University, Incheon 21936, Republic of Korea; woolim@gachon.ac.kr

**Keywords:** shoulder girdle muscle, electromyography, Pilates-based exercise, muscle activity ratio

## Abstract

Among the Pilates-based exercises, the modified side-arm (MSA) and modified high-five (MHF) are commonly used for shoulder strengthening and rehabilitation. This study examined shoulder girdle muscle activity and ratios across different spring intensities. Twenty-two healthy males performed the MSA and MHF using yellow (low), blue (medium), and red (high) springs. Surface electromyography (EMG) was used to measure serratus anterior (SA), lower trapezius (LT), levator scapulae (LS), upper trapezius (UT), and middle deltoid (MD) muscle activity, along with LS/SA, LS/LT, and UT/LT ratios during concentric, isometric, and eccentric phases. Muscle activities were generally higher in the MHF than in the MSA with the same spring. Both exercises demonstrated a proportional increase in activity with spring intensity, though the activity of the SA and LT in the MHF plateaued. MHF ratios were significantly higher with the red spring. These findings indicate that the MHF stimulates shoulder girdle muscles more than the MSA, and that the MSA can further stimulate shoulder girdle muscles by increasing spring intensity. Additionally, optimal spring intensity exists in the MHF for targeting shoulder stabilization muscles. However, excessive spring intensity during the MHF may lead to abnormal compensation, emphasizing the need for careful spring intensity progression.

## 1. Introduction

Pilates has been widely recognized as an exercise for enhancing overall body strength and flexibility since it was developed in the 1920s [1]. This exercise utilizes concentric, isometric, and eccentric contractions that require precise and controlled movements to correct muscle imbalances [2,3]. Recent research has highlighted the significant benefits of Pilates exercises in rehabilitation, making it a valuable tool in various clinical settings [2,4,5,6]. Specifically, Pilates-based shoulder exercises have shown promise for improving shoulder function, including range of motion (ROM), strength, flexibility, muscle balance, posture, and pain reduction [4,7,8,9].

Among the Pilates-based shoulder exercises, side-arm and high-five exercises involve shoulder abduction, a movement commonly used in rehabilitation and strength programs to enhance shoulder girdle muscle strength, stability, and balance [10,11,12,13]. In particular, shoulder abduction exercises play a crucial role in the rehabilitation of musculoskeletal disorders of the neck and shoulder. This is because a decrease in the upward rotation of the scapulothoracic cage leads to increased compression of the rotator cuff and tension in the cervicoscapular muscles, which can cause shoulder and neck pain. Therefore, inducing upward rotation through shoulder abduction exercises is essential [14,15,16]. However, the original side-arm and high-five exercises are performed in a high-kneeling position, which, in some cases, may excessively engage the core muscles, limiting the focus on lemeshoulder abduction. To address this limitation, both exercises were modified into a sitting position, allowing for greater emphasis on shoulder abduction. As a result, the modified side-arm (MSA) and modified high-five (MHF) serve as more efficient Pilates-based shoulder exercises [17,18,19,20,21].

The MSA and MHF are performed against a given spring intensity, similar to resistance exercises, in which muscle activity increases with load intensity. However, when the load intensity surpasses a certain threshold, muscle activity may plateau or decrease, depending on the muscle involved [10,22,23,24,25]. This phenomenon highlights that there is an optimal load intensity for maximizing the activation of each muscle, rather than simply increasing the load intensity. However, the relationship between spring intensity and muscle activity during the MSA and MHF remains unclear, limiting their precise application. Consequently, examining changes in shoulder girdle muscle activity with varying spring intensity during the MSA and MHF can help determine the appropriate spring intensity for maximizing the activation of the target shoulder girdle muscle, facilitating more effective clinical application.

Although both the MSA and MHF involve shoulder abduction up to 180°, they differ in their starting positions: MSA begins with the elbow flexed at 90°, whereas MHF starts with the elbow fully extended. Previous studies reported that even when performing the same shoulder movements at identical load intensities, shoulder girdle muscle activity varies depending on whether the elbow is flexed [26,27,28,29]. These differences suggest that the MSA and MHF may elicit distinct muscle activities despite using the same shoulder movement and spring intensity. Nonetheless, research examining the differences in muscle activity between the MSA and MHF remains insufficient. Accordingly, a comparative analysis of the differences between the MSA and MHF can provide scientific evidence for selecting appropriate exercise methods depending on each patient’s rehabilitation stage (early, middle, and late phases) when applying Pilates exercises for shoulder rehabilitation.

The shoulder girdle muscles related to the MSA and MHF require a multidimensional approach that emphasizes the strengthening and balance of muscle activity for effective rehabilitation [18,20,28,30,31]. The shoulder girdle muscles activated during the MSA and MHF, including the serratus anterior (SA) and lower trapezius (LT), play critical roles in shoulder stabilization within the scapulohumeral rhythm [32,33]. Weakness in these muscles is associated with scapular dyskinesis [34], which can lead to complex shoulder disorders such as shoulder impingement, subacromial pain syndrome, and winging scapula [35,36,37]. Thus, SA and LT require targeted strengthening [32,38]. Simultaneously, the levator scapulae (LS) and upper trapezius (UT), which are activated together with the SA and LT, perform distinct functions in the scapulohumeral rhythm by slightly elevating the scapula [39,40,41]. As a result, unlike the SA and LT, excessive activity of the LS and UT causes scapular dyskinesis and reduces the subacromial space, leading to shoulder pain [32,42,43]. This is why careful consideration of the SA, LT, LS, and UT activity ratios is crucial during the MSA and MHF for effective rehabilitation [18,20,28,30,31]. Hence, it is important to investigate which spring intensity during the MSA and MHF allowed for an appropriate ratio that maximizes SA and LT activation while minimizing LS and UT activation.

Therefore, this study aimed to investigate how changes in spring intensity affect the shoulder girdle muscle activity and ratios during the MSA and MHF. Additionally, it aimed to compare the muscle activity and ratios of the MSA and MHF under the same spring intensity to identify differences between the two movements. These findings are expected to offer insights into optimizing these Pilates-based exercises for strengthening and rehabilitation of shoulder girdle muscles.

## 2. Materials and Methods

### 2.1. Study Design

This cross-sectional study was conducted with a single-group design. To minimize bias in the intervention order, a block randomization method with a block size of six was applied. The randomization sequence was generated using Microsoft Excel (RAND function) to ensure that each participant performed the six interventions (the MSA and MHF with yellow, blue, and red springs) in a randomized order. This study was approved by the Gachon University Institutional Review Board (approval IRB number: 1044396-202409-HR-152-01) and registered with the clinical research information service (CRIS) in compliance with the WHO guidelines (registration no. KCT0010032). All participants were fully informed of the experimental procedures, excluding the specific purpose of the study, and they provided written informed consent.

### 2.2. Participants

Participants were recruited via announcements on a bulletin board and in an online community at Gachon University in Incheon. A total of 26 individuals initially responded, 22 of whom ultimately met the inclusion criteria. The inclusion criteria were as follows: (1) healthy adults aged 20–30 years and (2) individuals with a minimum of six months of experience in Pilates. The exclusion criteria were as follows: (1) orthopedic or neurological disorders, (2) cardiopulmonary disorders, and (3) other disabilities.

The sample size was determined using G*Power software (version 3.1.9.2, University of Kiel, Germany) and repeated-measures ANOVA. In addition, an effect size (f) of 0.25, an alpha level of 0.05, a power of 0.8, one group, and six measurements were entered, yielding the required sample size of 19. To accommodate an estimated dropout rate of 10% throughout the study, 22 participants were included in the final sample.

### 2.3. Procedure

The participants visited the integrated movement science laboratory (IMSL) at Gachon university over two days for the experiment. During the first visit, anthropometric measurements were conducted and familiarization sessions were provided to help participants understand the experimental procedures of the main measurement. The participants’ height, weight, BMI, body fat percentage, and skeletal muscle mass were assessed using a BMS330 (Biospace Co., Ltd., Seoul, Republic of Korea). During the second visit, the main measurements were conducted, including the assessment of maximum voluntary isometric contraction (MVIC) and muscle activity by using surface EMG during the exercises.

### 2.4. Intervention

The subjects performed MSA, MHF, and shoulder abduction exercises using a Pilates reformer (Into Pilates Inc., Seongnam, Republic of Korea). Both exercises were performed in three phases: concentric, isometric, and eccentric contractions. Each phase was performed for 2 s, and the exercises were synchronized with a metronome set to 60 bpm to control the execution time. The spring intensities of the reformer were adjusted to three levels: yellow (low intensity), blue (medium intensity), and red (high intensity), which are typically used for the early, middle, and late rehabilitation phases, respectively. Participants performed one repetition per set, completing three sets for each of the three spring intensities in the MSA and MHF. Before each set begins, one practice repetition is conducted. To reduce fatigue, participants were given a 5 min rest period between all sets and practice.

The intensity of the springs increased in the order of yellow (initial tension: 1.9–2 kg, spring rate: 0.33 kg/cm ± 5%), blue (initial tension: 3–3.2 kg, spring rate: 0.67 kg/cm ± 5%), and red (initial tension: 5.3–5.6 kg, spring rate: 0.88 kg/cm ± 5%). Under all conditions, both exercises began at a position in which the spring was stretched by 2 cm. The spring lengths increased by 23.5 cm (±2.6 cm) in the MSA and 53.8 cm (±4.1 cm) in the MHF.

To prevent errors in both exercises, familiarization was conducted one week prior, and detailed descriptions of this procedure are provided below. Additionally, the participants performed the exercises while looking at a mirror to monitor whether other parts of their bodies remained stable and whether the angles were correct. To ensure that the exercises were performed at the correct angles, the appropriate elbow and shoulder angles for the starting and ending positions were measured by goniometer (Patterson Medical., Warrenville, IL, USA), and markers were placed at corresponding positions on the mirror. Participants were instructed to perform the exercises only between these markers, ensuring that all participants executed the exercises at the correct angles. Simultaneously, the researchers continuously observed movement errors. If an error occurred, the data collected from that attempt were discarded, and the measurement was repeated.

#### 2.4.1. Modified Side-Arm

**Figure 1:**
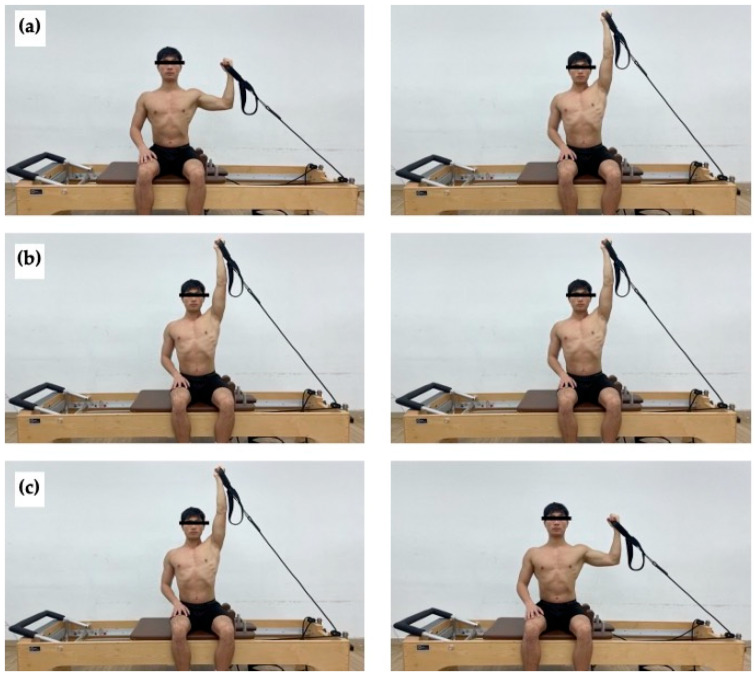
Modified side-arm (MSA) exercise. (**a**) Starting position (**left**) and end position (**right**) of concentric phase. (**b**) Starting position (**left**) and end position (**right**) of isometric phase. (**c**) Starting position (**left**) and end position (**right**) of eccentric phase.

MSA began in a seated position on the reformer. When the participant sat on the reformer, their thighs were positioned in contact with the shoulder rests, and the ASIS was aligned between the two shoulder rests to standardize the sitting position. The participants held the strap attached to the spring with their dominant hand, with the starting position consisting of 90° of shoulder abduction and elbow flexion, and with the palm facing their face, while the opposite hand rested on the thigh. In the concentric phase, the dominant arm was raised to 180° of shoulder abduction while fully extending the elbow against each spring intensity. Posture was maintained in the isometric phase. In the eccentric phase, the arm returned to the starting position of the concentric phase. During the exercise, all body parts except for the dominant arm were kept stationary (Figure 1).

#### 2.4.2. Modified High-Five

**Figure 2:**
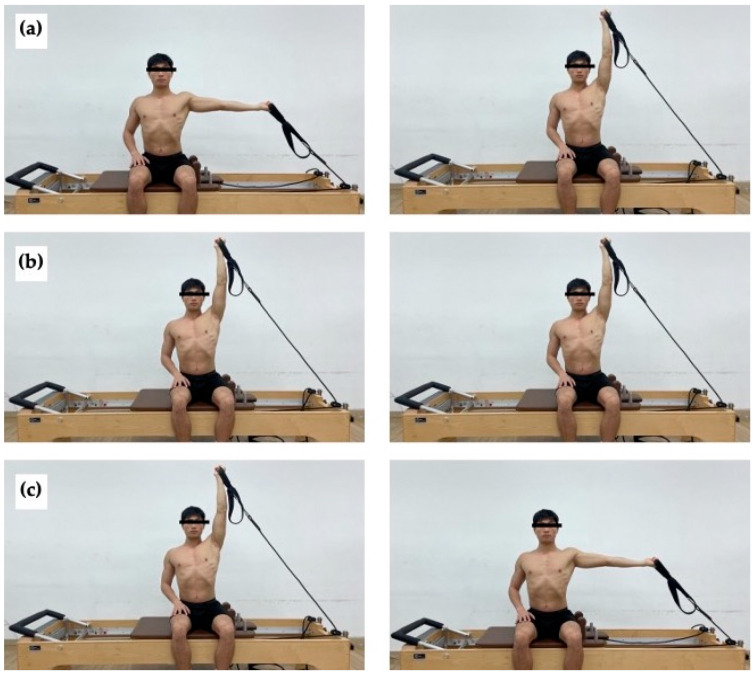
Modified high-five (MHF) exercise. (**a**) Starting position (**left**) and end position (**right**) of concentric phase. (**b**) Starting position (**left**) and end position (**right**) of isometric phase. (**c**) Starting position (**left**) and End position (**right**) of eccentric phase.

The MHF was performed in the same seated position on the reformer using the dominant arm. The exercise started with the dominant arm at 90° of shoulder abduction and the elbow fully extended with the palm facing upward. During the concentric phase, the arm was raised to 180° of shoulder abduction while maintaining a straight elbow. This posture was maintained in the isometric phase, while in the eccentric phase, the arm was returned to the starting position of the concentric phase. Similarly, all other body parts except for the dominant arm remained stationary throughout the exercise (Figure 2).

#### 2.4.3. Familiarization

A familiarization session was conducted during the first visit to the laboratory to familiarize the participants with the experimental procedures for the main measurement. In this session, the participants were introduced to the MSA and MHF exercises, as well as the process of dividing the measurements into concentric, isometric, and eccentric phases. They were also instructed to perform each phase for 2 s in sync with a metronome to ensure a clear understanding of the main measurement procedure.

Following the instructions, participants practiced the experimental procedure directly. During the familiarization session, they rehearsed the proper sitting positions, starting postures, movement angles, and timing using a metronome. Similarly to the main measurement, the participants practiced while looking into a mirror, allowing them to monitor whether body parts other than their dominant arm remained stable and whether the angles were properly formed. Markers were also attached to the corresponding positions on the mirror by measuring the elbow and shoulder angles in the same manner as in the main measurement, ensuring that exercises were performed only within the designated marker range. Feedback was provided for any movement errors to help the participants perform the exercises perfectly. This practice consisted of one repetition per set, with three sets performed for each of the three spring intensities in the MSA and MHF. A 5 min rest period was provided between sets, consistent with the main measurement rest period. The familiarization session took approximately 120 min.

### 2.5. Electromyographic Data Processing and Analysis

In this study, we collected surface EMG data for the LS, UT, SA, LT, and middle deltoid (MD) muscles. All the data were collected using BIOPAC (MP160, Biopac Systems Inc., Goleta, CA, USA) equipment. The units of measurement were recorded in millivolts (mV), and the raw electromyographic (EMG) signal was band-pass-filtered between 30 and 500 Hz, full-wave-rectified, and sampled at a rate of 2000 Hz (CMRR: > 110 dB at 60 Hz, input impedance > 1012 Ω). The collected EMG signals were processed using root mean square (RMS) values. AcqKnowledge software (version 5.0; Biopac Systems, Inc., Goleta, CA, USA) was used for signal recording and raw data processing. Muscle activity was described as %MVIC, normalized by dividing the mean EMG data extracted during the exercises by the peak EMG data obtained during maximum voluntary isometric contraction (MVIC) (mean EMG/peak MVIC). Muscle activity ratios were described as the values obtained by dividing each %MVIC by another (%MVIC/%MVIC).

Electromyography (EMG) was performed according to a protocol standardized by the SENIAM guidelines and in previous studies. The MVIC was performed to normalize the raw EMG data. The MVIC was measured three times and retained for 5 s for each attempt. A rest period of 90 s was allowed between each attempt [44]. LS, UT, SA, and MD activity was assessed with the participants in a sitting position, while LT activity was assessed in a prone position. LS assessment was performed with the neck rotated ipsilaterally and the shoulder elevated [45]. UT assessment was performed with the neck rotated contralaterally and flexed ipsilaterally, and the shoulder elevated [46]. SA assessed was performed with the shoulder flexed at 135° [20,47]. LT assessment was measured with the shoulder abducted to 135° and the thumb facing upward [48].

The hair over the targeted muscles was shaved, and the skin was cleaned with alcohol before attaching the electrodes. Disposable 10 mm Ag/AgCl bipolar surface electrodes (Bio-Protech Inc., Wonju, Republic of Korea) were used. Electrodes were placed on the dominant side of each muscle belly. Reference electrodes were attached to the inferior angle of the scapula, the olecranon, and both sides of the acromion. The LS was attached at the intersection of C3-C4 along the line connecting the anterior margin of the UT to the posterior margin of the sternocleidomastoid. The UT was placed at the midpoint between C7 and the acromion, and the SA was placed at the intersection of the mid-axillary line and the 7th intercostal space. The LT was positioned between T7 and the scapular spine and the MD was positioned between the acromion and deltoid tubercle [2,49,50].

### 2.6. Statistical Analyses

Statistical analyses were conducted using SPSS software (version 28.0; SPSS Inc., Chicago, IL, USA). Continuous variables were expressed as mean ± standard deviation. The Shapiro–Wilk test was used to verify normality. A repeated-measures analysis of variance (ANOVA) was used for within-group comparisons. When significant differences were observed, Bonferroni post hoc analysis was performed. Statistical significance was set at *p* < 0.05.

## 3. Results

The general characteristics of the participants are listed in Table 1. This study included 22 healthy male participants aged 20–29 years.

### 3.1. Muscle Activity

#### 3.1.1. Muscle Activity in Concentric Phase

Muscle activity according to spring intensity in the concentric phase is presented in Table 2. Statistically significant differences in SA muscle activity were observed between exercises and among spring intensities (F = 6.985, *p* = 0.001, ηp^2^ = 0.250). According to the post hoc analysis, MSA red and MHF blue showed significantly higher activity than MSA blue and MSA yellow.

In LT muscle activity, statistically significant differences were observed between exercises and among spring intensities (F = 46.925, *p* < 0.001, ηp^2^ = 0.691). According to the post hoc analysis, MHF red and MHF blue showed significantly higher activity than all spring intensities of MSA. MSA red showed significantly higher activity than MSA blue and MSA yellow. MSA blue showed significantly higher activity than MSA yellow.

In LS muscle activity, statistically significant differences were observed between exercises and among spring intensities (F = 64.364, *p* < 0.001, ηp^2^ = 0.754). According to the post hoc analysis, MHF red showed significantly higher activity than MHF blue, MHF yellow, and all the spring intensities of the MSA. MHF blue exhibited significantly higher activity than MHF yellow and all the spring intensities of the MSA. MHF yellow and MSA red showed significantly higher activities than MSA blue and MSA yellow, whereas MSA blue showed significantly higher activity than MSA yellow.

In UT muscle activity, statistically significant differences were observed between exercises and among spring intensities (F = 40.040, *p* < 0.001, ηp^2^ = 0.656). According to the post hoc analysis, MHF red showed significantly higher activity than MHF blue, MHF yellow, and all the spring intensities of MSA. MHF blue exhibited significantly higher activity than MHF yellow and all the spring intensities of the MSA. MHF yellow and MSA red showed significantly higher activity than MSA blue and MSA yellow, whereas MSA blue showed significantly higher activity than MSA yellow.

Finally, statistically significant differences in MD muscle activity were observed between exercises and among spring intensities (F = 57.164, *p* < 0.001, ηp^2^ = 0.731). According to the post hoc analysis, MHF red showed significantly higher activity than MHF blue, MHF yellow, and all the spring intensities of the MSA. MHF blue exhibited significantly higher activity than MHF yellow and all the spring intensities of the MSA. MHF yellow and MSA red showed significantly higher activities than MSA blue and MSA yellow, whereas MSA blue showed significantly higher activity than MSA yellow.

#### 3.1.2. Muscle Activity in the Isometric Phase

Muscle activity according to spring intensity in the isometric phase is shown in Table 3. Statistically significant differences in SA muscle activity were observed between exercises and among spring intensities (F = 12.953, *p* < 0.001, ηp^2^ = 0.382). The post hoc analysis revealed that MHF red showed significantly higher activity than MHF yellow, MSA blue, and MSA yellow. MHF blue showed significantly higher activity than MHF yellow and MSA yellow. MSA red was significantly higher than MSA blue and MSA yellow, whereas MSA blue was significantly higher than MSA yellow.

In LT muscle activity, statistically significant differences were observed between exercises and among spring intensities (F = 38.714, *p* < 0.001, ηp^2^ = 0.648). According to the post hoc analysis, MHF red showed significantly higher activity than MHF blue, MHF yellow, and all the spring intensities of the MSA. MHF blue showed significantly higher activity than MHF yellow, MSA blue, and MSA yellow. MSA red showed significantly higher activity than MSA blue and MSA yellow, whereas MSA blue showed significantly higher activity than MSA yellow.

In LS muscle activity, statistically significant differences were observed between exercises and among spring intensities (F = 37.856, *p* < 0.001, ηp^2^ = 0.643). According to the post hoc analysis, MHF red showed significantly higher activity than MHF blue, MHF yellow, and all the MSA intensities. MHF blue showed significantly higher activity than MHF yellow, MSA blue, and MSA yellow, whereas MHF yellow showed significantly higher activity than MSA yellow. Additionally, MSA red activity was significantly higher than MSA blue and MSA yellow activity, whereas MSA blue activity was significantly higher than MSA yellow activity.

In UT muscle activity, statistically significant differences were observed between exercises and among spring intensities (F = 21.969, *p* < 0.001, ηp^2^ = 0.511). According to the post hoc analysis, MHF red showed significantly higher activity than MHF blue, MHF yellow, and all the spring intensities of MSA. MHF blue showed significantly higher activity than MHF yellow and MSA yellow. MSA red activity was significantly higher than MSA blue and MSA yellow activity, whereas MSA blue activity was significantly higher than MSA yellow activity.

Finally, statistically significant differences in MD muscle activity were observed between exercises and among spring intensities (F = 30.865, *p* < 0.001, ηp^2^ = 0.595). According to the post hoc analysis, MHF red showed significantly higher activity than MHF blue and MHF yellow, as well as all the MSA intensities, and MHF blue showed significantly higher activity than MHF yellow and MSA yellow. MSA red showed significantly higher activity than MHF yellow, MSA blue, and MSA yellow, whereas MSA blue showed significantly higher activity than MSA yellow.

#### 3.1.3. Muscle Activity in Eccentric Phase

Muscle activity according to spring intensity in the eccentric phase is described in Table 4. Statistically significant differences in SA muscle activity were observed between exercises and among spring intensities (F = 46.301, *p* < 0.001, ηp^2^ = 0.688). The post hoc analysis revealed that MHF red showed significantly higher activity than MHF blue, MHF yellow, and all the spring intensities of the MSA. MHF blue and MSA red showed significantly higher activity than MHF yellow, MSA blue, and MSA yellow. MHF yellow and MSA blue showed significantly higher activity than MSA yellow.

In LT muscle activity, statistically significant differences were observed between exercises and among spring intensities (F = 25.414, *p* < 0.001, ηp^2^ = 0.548). According to the post hoc analysis, MHF red showed significantly higher activity than MHF blue, MHF yellow, and all the spring intensities of MSA. MHF blue showed significantly higher activity than MHF yellow, MSA blue, and MSA yellow. MSA red showed significantly higher activity than MSA yellow.

In LS muscle activity, statistically significant differences were observed between exercises and among spring intensities (F = 58.343, *p* < 0.001, ηp^2^ = 0.735). According to the post hoc analysis, MHF red showed significantly higher activity than MHF blue, MHF yellow, and all the spring intensities of MSA. MHF blue and MSA red showed significantly higher activity than that of MHF yellow, MSA blue, and MSA yellow. MHF yellow and MSA blue showed significantly higher activity than MSA yellow.

In UT muscle activity, statistically significant differences were observed between exercises and among spring intensities (F = 36.984, *p* < 0.001, ηp^2^ = 0.638). According to the post hoc analysis, MHF red showed significantly higher activity than MHF blue, MHF yellow, and all the MSA intensities. MHF red showed significantly higher activity than MSA blue and MSA yellow.

Finally, statistically significant differences in MD muscle activity were observed between exercises and among spring intensities (F = 58.843, *p* < 0.001, ηp^2^ = 0.737). According to the post hoc analysis, MHF red showed significantly higher activity than MHF blue, MHF yellow, and all the spring intensities of MSA. MHF blue showed significantly higher activity than MHF yellow, MSA blue, and MSA yellow. MHF yellow and MSA blue showed significantly higher activity than MSA yellow, whereas MSA red showed significantly higher activity than MHF yellow and MSA yellow.

### 3.2. Muscle Activity Ratio

#### 3.2.1. Muscle Activity Ratios in Concentric Phase

The muscle activity ratios according to spring intensity in the concentric phase are presented in Table 5. First, the LS/SA ratio showed statistically significant differences between exercises and among spring intensities (F = 36.999, *p* < 0.001, ηp^2^ = 0.638). According to the post hoc analysis, the LS/SA ratio in MHF red was significantly higher than those in MHF blue, MHF yellow, and all the spring intensities of MSA. The LS/SA ratio in MHF blue was significantly higher than those in MHF yellow and all the spring intensities of the MSA.

The LS/LT ratio also showed statistically significant differences between exercises and among spring intensities (F = 11.253, *p* < 0.001, ηp^2^ = 0.360). The post hoc analysis revealed that the LS/LT ratios in MHF red was significantly higher than those in MHF blue, MHF yellow, MSA blue, and MSA yellow. The LS/LT ratio in MSA red was significantly higher than that in MSA blue.

On the other hand, no statistically significant differences were observed in the UT/LT ratio between exercises and among spring intensities (F = 0.830, *p* = 0.475, ηp^2^ = 0.042).

#### 3.2.2. Muscle Activity Ratios in Isometric Phase

The muscle activity ratios according to spring intensity in the isometric phase are described in Table 6. Firstly, statistically significant differences in LS/SA ratio were observed between exercises and among spring intensities (F = 6.407, *p* = 0.001, ηp^2^ = 0.234). According to the post hoc analysis, the LS/SA ratio was significantly higher in MHF red than in MHF blue, MHF yellow, and MSA blue.

The LS/LT ratio showed statistically significant differences between exercises and among spring intensities (F = 6.407, *p* < 0.001, ηp^2^ = 0.234). The LS/LT ratio in MHF red was significantly higher than those in MHF blue, MHF yellow, and MSA blue.

In the UT/LT ratio, no statistically significant differences were observed between exercises and among spring intensities (F = 0.190, *p* = 0.902, ηp^2^ = 0.009).

#### 3.2.3. Muscle Activity Ratios in Eccentric Phase

The muscle activity ratios according to spring intensity in the eccentric phase are described in Table 7. Statistically significant differences in LS/SA ratios were observed between exercises and among spring intensities (F = 6.704, *p* < 0.001, ηp^2^ = 0.251). According to the post hoc analysis, the LS/SA ratio in MHF red was significantly higher than those in MHF yellow and MSA blue.

The LS/LT ratio showed statistically significant differences between exercises and among spring intensities (F = 9.578, *p* < 0.001, ηp^2^ = 0.324). The post hoc analysis revealed that the LS/LT ratio in MHF red was significantly higher than those in MHF blue, MHF yellow, MSA blue, and MSA yellow.

The UT/LT ratio also showed statistically significant differences between exercises and among spring intensities (F = 7.516, *p* < 0.001, ηp^2^ = 0.273). The post hoc analysis indicated that the UT/LT ratio in MSA red was significantly higher than those in MHF yellow and MSA yellow. The UT/LT ratios for MHF blue and MSA red were significantly higher than that for MSA yellow.

## 4. Discussion

This study identified shoulder girdle muscle activity and abnormal compensation generated by the MSA and MHF among different spring intensities (yellow, blue, and red springs). Muscle activity was generally higher in the MHF than in the MSA in the same spring. MSA and MHF muscle activities tended to increase proportionally with spring intensity, although plateaus were observed only in the SA and LT of the MHF. These results indicate that the shoulder girdle muscles can be stimulated more through MHF than through the MSA and that the MSA can further stimulate the shoulder girdle muscles by increasing the spring intensity. However, the findings also suggest that there is an optimal spring intensity in the MHF for selectively targeting and strengthening only the shoulder stabilization muscles (SA, LT). The LS/SA, LS/LT, and UT/LT ratios between the MSA and MHF in the same spring generally showed no significant differences, and the MSA ratios among the spring intensities also showed no significant differences. Conversely, the ratios of the MHF among the spring intensities tended to be significantly higher in red, which represents the highest spring intensity. This suggests that excessive increases in spring intensity during the MHF significantly enhance the compensatory muscles rather than the shoulder stabilization muscles. This indicates the need for caution when increasing the spring intensity in the MHF exercises.

The MSA and MHF are Pilates-based exercises that involve shoulder abduction of up to 180°. However, even when the two exercises were performed using the same spring, muscle activity showed a significantly higher tendency during the MHF than during the MSA across all phases. Campos et al. compared the muscle activities of two exercises, the shoulder press and lateral raise, both of which involve shoulder abduction, whereas Solstad et al. compared the muscle activities of the bench press and dumbbell press, both of which involve shoulder horizontal adduction [26,29]. In these prior studies, significant differences in muscle activity were observed between the two exercises, even when performed under the same load intensity. These findings are consistent with those of the current study, which revealed differences in muscle activity depending on the exercise (the MSA or MHF), despite the participants using the same spring [26,29]. Although the MSA and MHF involve the same shoulder movements, they differ significantly in their biomechanical characteristics. The MSA begins with the elbow flexed at 90°, whereas the MHF starts with the elbow fully extended. Consequently, the MHF has a longer external moment arm, and the spring length increases more than twice as much in the MHF (53.8 ± 2.6 cm) compared to MSA (23.5 cm ± 2.6). As the external moment arm increases in the MHF, the torque required to lift the arm against the spring resistance is also greater than in the MSA. This occurs because torque is the product of force and the perpendicular distance from the axis of rotation to the line of action of force [51,52]. Additionally, as the spring stretches further in the MHF compared to the MSA, the restoring force exerted by the spring is also stronger in the MHF, in accordance with Hooke’s Law, which states that force is proportional to the amount of spring stretch. This increased restoring force results in greater resistance in the MHF compared to the MSA [53,54]. These principles indicate that the MHF is performed at a higher spring intensity than the MSA, even when using the same spring. Because higher intensity leads to increased motor unit recruitment and firing rates, low-load intensity recruits fewer motor units compared to high-load intensity. When more motor units are recruited, electrical signals increase, leading to higher EMG data. Thus, several studies have shown that muscle activity generally increases as load intensity rises [23,24,55,56,57]. For these reasons, MHF muscle activities are considered to be higher than MSA activity. Given this, since the MSA is relatively easier to perform than the MHF, it can be incorporated in the earlier phases of rehabilitation, where lower intensity is required. As rehabilitation progresses and a higher level of muscle activity becomes necessary, the MHF can be introduced as a more challenging exercise.

The MSA and MHF were performed against the following spring intensities: yellow (low level), blue (medium level), and red (high level). According to the findings of this study, all MSA muscle activities showed a proportional increase with an increase in spring intensity across all phases. Notably, this trend was particularly pronounced in the MD. However, the increases in SA and LT in the MHF were not significant as the spring intensities increased, and SA even showed a decrease under the red spring, forming a plateau. Several authors have compared muscle activity during resistance exercises at different load intensities and reported results consistent with the trend observed in the MSA, showing a proportional increase in muscle activity with load intensities up to 70% of the 1RM [23,24,25,55,56,57]. Interestingly, beyond 70% of the 1RM, muscle activity plateaued, similar to the trends observed in the MHF [24,25]. Tsoukos et al. observed muscle activity during resistance exercises at different load intensities exceeding 40% of the 1RM [24]. Their study indicated that while muscle activity increased significantly in proportion to load intensities, no significant differences were found beyond 70% of the 1RM [24]. Similarly, Vigotsky et al. compared muscle activity across load intensities ranging from 50% to 90% of the 1RM and reported proportional increases in muscle activity up to 70% of the 1RM. However, beyond 70% of the 1RM, some muscle activity begins to decrease [25]. In general, muscle activity is reported to be higher under high-load intensities than under low-load intensities as motor unit recruitment increases with increasing load intensity [23,24,55,56,57]. As a result, the muscle activity of the MSA in this study also appeared to increase proportionally with spring intensity. In contrast, trends similar to those of MHF muscle activity are commonly observed at extremely high load intensities, such as 70–90% of the 1RM or 80% of the MVC [24,25,58]. This phenomenon occurs because the motor units are already recruited to nearly maximum levels at a given load intensity, and further increases in the load intensity fail to recruit additional motor units [58]. Considering that the MHF is performed at a higher intensity than the MSA because of differences in movement characteristics [51,52,53,54], it is inferred that the MHF does not recruit additional motor units even with increased spring intensity, resulting in a plateau. These findings suggest that, in clinical applications, increasing spring intensity during the MSA can be an effective strategy to enhance the activation of shoulder girdle muscles. However, in the case of the MHF, increasing spring intensity has limited effects on the activation of shoulder stabilization muscles, such as SA and LT. Therefore, alternative strategies, such as increasing the number of repetitions, may be considered to achieve intended muscle activity. Previous studies have primarily demonstrated these findings in resistance exercises commonly used for muscle strengthening. However, our study expands this evidence to Pilates exercises, confirming their applicability. Thus, this study highlights the potential for direct implementation of these Pilates exercises in clinical shoulder rehabilitation.

This study examined the LS/SA, LS/LT, and UT/LT ratios to observe how the activation balance of the shoulder girdle muscles changes with different spring intensities during the MSA and MHF, which involve scapular upward rotation. The SA, LT, LS, and UT muscles were activated simultaneously during the upward rotation. Among these, the SA and LT function as shoulder stabilizers [32,33], and their weakness can lead to complex shoulder disorders [34,35,36,37]. Therefore, these muscles require targeted strengthening during both exercises [32,38]. However, the LS and UT slightly elevate the shoulder during scapular upward rotation [39,40,41]. In contrast to the SA and LT, excessive activation of these muscles can cause scapular dyskinesis and reduce the subacromial space, leading to shoulder pain. Furthermore, chronic overactivation of compensatory muscles may lead to persistent postural dysfunctions, particularly upper crossed syndrome, which is characterized by muscular imbalances, resulting in mechanical strain [32,42,43]. Thus, to maintain shoulder girdle muscle balance, exercises must be performed to prevent significant increases in each ratio [18,20,30,31].

In this study, the ratios in the MHF tended to be significantly higher with the red spring compared to the yellow and blue springs. Kim et al. analyzed shoulder girdle muscle activity ratios during abduction exercises at 20%, 50%, and 75% load intensities [18]. They reported significantly different ratios at the highest intensity, indicating an imbalance between stabilizing and compensatory muscles, aligning with the trends observed in the MHF. Prior studies have suggested that the highest load intensity impairs motor control, leading to imbalanced ratios [18,59]. Likewise, the highest intensity (red spring) in this study was likely associated with reduced motor control efficiency during the MHF, resulting in significantly higher ratios. Therefore, practitioners should exercise caution when increasing spring intensity during the MHF to prevent abnormal compensation and shoulder pain.

The ratios in the MSA generally did not show significant differences among the spring intensities. According to previous studies, a load intensity as high as 75% is required for the motor control efficiency to decrease sufficiently to result in significant differences in the ratios [18]. Because of the inherent differences between the MHF and MSA, the MSA is performed at much lower spring intensities compared to the MHF [51,52,53,54]. Therefore, even with the application of the red spring, MSA did not generate a sufficiently high intensity to cause a significant drop in motor control efficiency, which likely explains the general lack of significant differences observed in the ratios for the MSA.

When the MSA and MHF, which involve the same shoulder movements, were performed using the same spring, the MHF ratios were higher across all phases. However, most differences were not statistically significant. Kara et al. compared shoulder girdle muscle activity ratios during exercises with the same shoulder movements using the same resistance band at 0° and 90° elbow flexion with the same shoulder abduction angle [28]. They reported no significant differences, which is consistent with the findings of this study [28]. However, significant differences were observed when comparing the shoulder girdle muscle ratios of exercises involving the same shoulder movements at different shoulder abduction angles, which is inconsistent with the results of this study [27,28]. These inconsistent findings may be because shoulder girdle muscle activity ratios are more closely related to shoulder angle than to elbow angle. Although the MSA and MHF are performed with different elbow flexion angles, since they are performed within the same range of shoulder abduction angles, the tendency for no significant differences in the shoulder girdle muscle activity ratios observed in this study seems reasonable.

This study has several limitations. First, we recruited a small sample of 22 healthy adult males in their 20s. This makes it difficult to generalize the findings to individuals of different sexes, ages, or specific medical conditions. Consequently, future research is needed to include populations of different age groups and sexes. In particular, individuals with certain orthopedic disorders may exhibit differences in range of motion (ROM) compared to healthy participants. Accordingly, it is recommended that future studies divide movement ranges and assess muscle activity within each specific range. Second, this study recruited participants with approximately one year of Pilates experience, which may limit the generalizability of the findings to highly experienced individuals. Future research should investigate whether the optimal spring intensities identified in this study apply to individuals with advanced practitioners. Finally, only SA, LT, LS, UT, MD muscles were measured. Therefore, future research should analyze additional muscle activities and ratios for other shoulder girdle muscles and core muscles.

## 5. Conclusions

The conclusions of this study are as follows. First, the activity of the shoulder girdle muscles during the MSA and MHF does not necessarily increase proportionally with the spring intensity, indicating that each target muscle has an optimal spring intensity to maximize activation. Notably, the ratios representing compensatory muscles (LS and UT) relative to shoulder stabilizing muscles (SA and LT) were higher when the MHF was performed with the red spring. This suggests that appropriate spring application is essential to effectively stimulate the desired muscles during each exercise, while simultaneously considering excessive progression in spring intensity to avoid muscle imbalance during the MHF. Particularly, when performing the MHF with a high-level spring (red), the activation of shoulder stabilizing muscles decreases, while the ratio of compensatory muscles becomes significantly imbalanced. Therefore, it is recommended to progress only up to a medium-level spring intensity.

Second, shoulder girdle muscle activity was significantly higher in the MHF than in the MSA when performed in the same spring; however, no differences were observed in the ratios. This implies that selecting a spring suitable for the participant’s characteristics among low-, medium-, and high-level springs and subsequently progressing from the MSA to the MHF based on the individual rehabilitation stage may be an effective strategy.

Furthermore, unlike previous studies that did not distinguish between concentric and eccentric contraction EMG data, this study analyzed each phase separately. This approach allows for a detailed understanding of the muscle activation patterns of the MSA and MHF across each phase, thus providing a unique advantage.

## Figures and Tables

**Table 1:** General characteristics of the participants.

	Mean ± SD
Age (years)	22.88 ± 1.45
Height (cm)	173.69 ± 5.20
Mass (Kg)	77.31 ± 8.61
Body Mass index (kg/m^2^)	25.61 ± 2.43
Skeletal muscle mass	36.06 ± 3.41
Percent body fat	16.89 ± 5.17

SD: standard deviation.

**Table 2:** Muscle activity in concentric phase.

	Modified Side-Arm			Modified High-Five			F (*p*-Value)	ηp^2^	Post Hoc
	Yellow (a)	Blue (b)	Red (c)	Yellow (d)	Blue (e)	Red (f)			
	M ± SD	M ± SD	M ± SD	M ± SD	M ± SD	M ± SD			
SA	15.59 ± 5.56	16.20 ± 4.85	17.95 ± 5.18	17.06 ± 5.27	19.29 ± 5.84	19.22 ± 6.04	6.985 (*p* = 0.001)	0.250	ab < ce
LT	10.37 ± 4.86	12.05 ± 5.71	13.37 ± 5.79	13.79 ± 5.01	16.46 ± 5.57	18.04 ± 6.15	46.925 (*p* < 0.001)	0.691	a < b < c < ef
LS	6.04 ± 2.38	7.08 ± 2.96	9.08 ± 3.62	9.29 ± 3.76	12.50 ± 5.06	17.58 ± 6.08	64.364 (*p* < 0.001)	0.754	a < b < cd < e < f
UT	9.12 ± 6.30	10.32 ± 6.69	11.95 ± 7.04	12.91 ± 8.51	16.13 ± 8.37	18.38 ± 8.52	40.040 (*p* < 0.001)	0.656	a < b < cd < e < f
MD	14.33 ± 4.69	16.09 ± 4.17	18.18 ± 4.70	17.61 ± 3.96	20.76 ± 4.99	22.68 ± 5.18	57.164 (*p* < 0.001)	0.731	a < b < cd < e < f

M: mean; SD: standard deviation; ηp^2^: partial eta squared; LS: levator scapulae; UT: upper trapezius; SA: serratus anterior; LT: lower trapezius; MD: middle deltoid.

**Table 3:** Muscle activity in isometric phase.

	Modified Side-Arm			Modified High-Five			F (*p*-Value)	ηp^2^	Post Hoc
	Yellow (a)	Blue (b)	Red (c)	Yellow (d)	Blue (e)	Red (f)			
	M ± SD	M ± SD	M ± SD	M ± SD	M ± SD	M ± SD			
SA	18.08 ± 7.38	21.60 ± 8.62	24.04 ± 9.63	20.97 ± 9.28	24.65 ± 10.04	27.34 ± 9.59	12.953 (*p* < 0.001)	0.382	ad < ef a < b < cf
LT	9.83 ± 5.08	12.09 ± 5.69	14.19 ± 6.18	12.01 ± 4.75	15.84 ± 6.32	18.76 ± 6.38	38.714 (*p* < 0.001)	0.648	a < b<ce < f d < e < f
LS	9.50 ± 3.88	11.14 ± 4.68	13.42 ± 5.23	11.67 ± 4.92	14.99 ± 6.32	20.43 ± 7.94	37.856 (*p* < 0.001)	0.643	a < b < ce < f a < d < e < f
UT	12.05 ± 7.93	13.94 ± 8.23	15.74 ± 8.37	14.23 ± 8.58	18.42 ± 8.77	22.13 ± 8.81	21.969 (*p* < 0.001)	0.511	a < b < c < f d < e < f a < e
MD	19.08 ± 5.63	22.63 ± 5.77	25.81 ± 5.80	20.69 ± 5.86	25.95 ± 7.36	28.24 ± 7.77	30.865 (*p* < 0.001)	0.595	a < b < c < f ad < e < f d < c

M: mean; SD: standard deviation; ηp^2^: partial eta squared; LS: levator scapulae; UT: upper trapezius; SA: serratus anterior; LT: lower trapezius; MD: middle deltoid.

**Table 4:** Muscle activities in eccentric phase.

	Modified Side-Arm			Modified High-Five			F (*p*-Value)	ηp^2^	Post Hoc
	Yellow (a)	Blue (b)	Red (c)	Yellow (d)	Blue (e)	Red (f)			
	M ± SD	M ± SD	M ± SD	M ± SD	M ± SD	M ± SD			
SA	8.19 ± 3.31	11.14 ± 4.47	13.20 ± 5.29	10.18 ± 4.01	14.37 ± 4.89	17.99 ± 5.04	46.301 (*p* < 0.001)	0.688	a < bd < ce < f
LT	8.58 ± 4.81	9.49 ± 5.38	10.55 ± 5.05	8.70 ± 4.61	12.29 ± 5.68	14.28 ± 6.07	25.414 (*p* < 0.001)	0.548	a < ce < f bd < e < f
LS	4.10 ± 1.46	5.44 ± 2.37	7.16 ± 3.44	5.20 ± 2.03	7.82 ± 3.33	11.42 ± 4.16	58.343 (*p* < 0.001)	0.735	a < bd < ce < f
UT	5.22 ± 3.64	6.82 ± 4.32	8.27 ± 5.26	6.44 ± 4.19	10.03 ± 5.50	13.03 ± 6.15	36.984 (*p* < 0.001)	0.638	a < b < ce < f d < e < f
MD	7.81 ± 2.74	10.75 ± 3.62	12.13 ± 3.67	9.87 ± 2.92	14.20 ± 4.10	17.34 ± 4.20	58.843 (*p* < 0.001)	0.737	a < bd < e < f a < d < c < f

M: mean; SD: standard deviation; ηp^2^: partial eta squared; LS: levator scapulae; UT: upper trapezius; SA: serratus anterior; LT: lower trapezius; MD: middle deltoid.

**Table 5:** Muscle activity ratios in concentric phase.

	Modified Side-Arm			Modified High-Five			F (*p*-Value)	ηp^2^	Post Hoc
	Yellow (a)	Blue (b)	Red (c)	Yellow (d)	Blue (e)	Red (f)			
	M ± SD	M ± SD	M ± SD	M ± SD	M ± SD	M ± SD			
LS/SA	0.50 ± 0.32	0.54 ± 0.29	0.60 ± 0.29	0.60 ± 0.28	0.73 ± 0.30	0.99 ± 0.37	36.999 (*p* < 0.001)	0.638	abcd < e < f
LS/LT	0.70 ± 0.41	0.70 ± 0.40	0.78 ± 0.42	0.72 ± 0.35	0.84 ± 0.40	1.03 ± 0.44	11.253 (*p* < 0.001)	0.360	ade < f b < cf
UT/LT	1.04 ± 0.82	0.96 ± 0.65	0.96 ± 0.61	1.00 ± 0.76	1.01 ± 0.63	1.08 ± 0.71	0.830 (*p* = 0.475)	0.042	-

M: mean; SD: standard deviation; ηp^2^: partial eta squared; LS: levator scapulae; UT: upper trapezius; SA: serratus anterior; LT: lower trapezius.

**Table 6:** Muscle activity ratios in isometric phase.

	Modified Side-Arm			Modified High-Five			F (*p*-Value)	ηp^2^	Post Hoc
	Yellow (a)	Blue (b)	Red (c)	Yellow (d)	Blue (e)	Red (f)			
	M ± SD	M ± SD	M ± SD	M ± SD	M ± SD	M ± SD			
LS/SA	0.52 ± 0.23	0.51 ± 0.23	0.57 ± 0.23	0.54 ± 0.23	0.56 ± 0.23	0.67 ± 0.23	6.407 (*p* = 0.001)	0.234	bde < f
LS/LT	0.52 ± 0.23	0.51 ± 0.25	0.57 ± 0.26	0.54 ± 0.28	0.56 ± 0.28	0.67 ± 0.31	6.407 (*p* < 0.001)	0.234	bde < f
UT/LT	1.13 ± 0.89	1.10 ± 0.79	1.10 ± 0.74	1.11 ± 0.81	1.15 ± 0.71	1.15 ± 0.67	0.190 (*p* = 0.902)	0.009	-

M: mean; SD: standard deviation; ηp^2^: partial eta squared; LS: levator scapulae; UT: upper trapezius; SA: serratus anterior; LT: lower trapezius.

**Table 7:** Muscle activity ratios in eccentric phase.

	Modified Side-Arm			Modified High-Five			F (*p*-Value)	ηp^2^	Post Hoc
	Yellow (a)	Blue (b)	Red (c)	Yellow (d)	Blue (e)	Red (f)			
	M ± SD	M ± SD	M ± SD	M ± SD	M ± SD	M ± SD			
LS/SA	0.53 ± 0.23	0.51 ± 0.25	0.57 ± 0.27	0.54 ± 0.28	0.56 ± 0.28	0.68 ± 0.32	6.704 (*p* < 0.001)	0.251	bd < f
LS/LT	0.50 ± 0.27	0.58 ± 0.34	0.67 ± 0.36	0.59 ± 0.30	0.65 ± 0.34	0.80 ± 0.38	9.578 (*p* < 0.001)	0.324	abde < f
UT/LT	0.57 ± 0.46	0.71 ± 0.55	0.78 ± 0.61	0.69 ± 0.56	0.84 ± 0.59	0.90 ± 0.59	7.516 (*p* < 0.001)	0.273	a < cef d < f

M: mean; SD: standard deviation; ηp^2^: partial eta squared; LS: levator scapulae; UT: upper trapezius; SA: serratus anterior; LT: lower trapezius.

## Data Availability

The data for this study are available from the corresponding authors on reasonable request.
